# Effect of music on anxiety among adolescents: a systematic review

**DOI:** 10.3389/fpsyg.2026.1822041

**Published:** 2026-07-15

**Authors:** Yingjie Lu, Joanne Pei Sze Yeoh, Kim Geok Soh, Xutao Liu

**Affiliations:** 1 Department of Music, Faculty of Human Ecology, Universiti Putra Malaysia, Serdang, Malaysia; 2 Department of Sports Studies, Faculty of Educational Studies, Universiti Putra Malaysia, Serdang, Malaysia; 3 School of Physical Education, Jiangsu University of Science and Technology, Zhenjiang, China

**Keywords:** adolescents, anxiety, emotion regulation, music interventions, psychology, systematic review

## Abstract

**Background:**

Adolescence is a developmental period marked by heightened vulnerability to anxiety, driven by academic pressure, social expectations, and rapid emotional changes. Music-based interventions have been examined as accessible, low-risk approaches for anxiety reduction; however, existing evidence remains conceptually fragmented and methodologically diverse.

**Methods:**

This systematic review followed PRISMA 2020 guidelines. A comprehensive search across PubMed, APA PsycINFO, Scopus, Web of Science, and CNKI identified randomized controlled trials (RCTs) and quasi-experimental studies examining the effects of music interventions on adolescent anxiety. Twenty studies (15 RCTs and 5 quasi-experiments) met the inclusion criteria. Data on study characteristics, intervention type, duration, frequency, and anxiety-related outcomes were extracted. Study quality was assessed using the PEDro scale.

**Results:**

Across 1,912 adolescents aged 12–18, most studies reported reductions in anxiety following music-based interventions, although the strength and durability of effects differed across intervention types. Active forms of music engagement (e.g., singing, instrument playing, improvisation) tended to produce more robust and sustained anxiety reduction compared with passive listening. Several studies also documented secondary improvements such as reduced depressive symptoms, enhanced stress regulation, and improved emotional well-being. Nonetheless, variations in intervention design, inconsistent reporting of musical features, and limited long-term follow-up constrained cross-study comparability.

**Conclusion:**

Music-based interventions appear beneficial for reducing anxiety among adolescents in both school and clinical contexts. Yet important gaps remain, particularly regarding the specification of musical elements, cultural adaptation of music selections, and integration with complementary psychological or school-based programs. Future work should employ more rigorous designs, include culturally grounded musical components, and systematically investigate combined intervention models and underlying mechanisms to strengthen the evidence base.

**Systematic review registration:**

CRD420251129625, https://www.crd.york.ac.uk/PROSPERO/view/CRD420251129625.

## 1 Introduction

Adolescence represents a formative developmental stage characterized by rapid physical, cognitive, and socio-emotional transitions. During this time, adolescents often encounter heightened academic demands, shifting peer relationships, and evolving identities, all of which contribute to increased vulnerability to anxiety (Steinberg, 2014). Epidemiological studies estimate that up to 20% of adolescents experience clinically significant anxiety symptoms (Polanczyk et al., 2015). This prevalence is concerning because untreated adolescent anxiety can impair academic performance, reduce social engagement, and elevate the risk of long-term mental health challenges, including depression and substance misuse (Copeland et al., 2014; Pine et al., 1998). In addition, untreated anxiety in adolescence imposes substantial economic and social costs, including increased healthcare utilization, reduced productivity, and long-term burden on public health systems (Trautmann et al., 2016). These findings underscore the need for practical, accessible interventions, particularly in school and community contexts where traditional clinical resources may be limited.

Increasingly, researchers and practitioners are exploring non-pharmacological interventions as scalable, low-risk options to support adolescent mental health. Among these, music-based interventions have garnered attention due to their accessibility, cultural adaptability, and potential to engage adolescents in a meaningful way. Music engages both emotional and cognitive domains, acting as a tool for distraction, a channel for emotional expression, and a method to regulate physiological arousal (Thoma et al., 2013). Neuroimaging studies confirm that music activates brain regions associated with emotion regulation and reward, including the limbic system and prefrontal cortex, providing a plausible mechanism for its anxiolytic effects (Koelsch, 2014; Zatorre and Salimpoor, 2013). Moreover, listening to calming music has been shown to decrease heart rate, autonomic arousal, and stress hormones, such as cortisol, all of which are markers associated with anxiety (Bernardi et al., 2006; Chanda and Levitin, 2013). Participatory music-making, such as group singing, drumming, or playing instruments, further enhances these effects by fostering social connection, self-expression, and a sense of mastery (Rickson and Watkins, 2003).

Empirical studies examining music’s impact on adolescent anxiety have yielded encouraging results. Meta-analyses and randomized controlled trials suggest moderate-to-large reductions in self-reported anxiety following music therapies or structured music programs (Bradt et al., 2013). These interventions span a variety of formats, including passive listening, active music creation, group singing, and songwriting. They are delivered across settings—from schools and community centers to hospitals and rehabilitation clinics. Despite this breadth of inquiry, the body of evidence is highly heterogeneous: intervention types, session duration, frequency, and measurement instruments vary widely. For example, some programs involve a single short listening session, while others run weekly over several months. Anxiety is often measured with different tools, such as the State–Trait Anxiety Inventory (STAI), Beck Anxiety Inventory (BAI), or visual analogue scales, which complicates direct comparisons of outcomes.

Moreover, many studies include adolescents with clinical or medical vulnerabilities (e.g., anxiety disorders, chronic illness, post-operative recovery), while others recruit healthy volunteer populations. Particularly, school-based interventions targeting general adolescent cohorts remain underrepresented, yet they are highly relevant for preventive mental health strategies (Rickson and McFerran, 2007). The lack of uniformity in populations, settings, and methodological rigor, such as small sample sizes, insufficient blinding, or short-term follow-up, further limits our ability to draw generalizable conclusions about music’s effectiveness for anxiety reduction in adolescents (Bradt et al., 2010; Gold et al., 2004).

Given these gaps, a focused synthesis of existing empirical findings is warranted. Although previous reviews have examined music interventions in clinical or mixed-age populations (Aalbers et al., 2017; Bradt et al., 2013), fewer have concentrated specifically on adolescents while simultaneously considering developmental factors that may shape intervention responsiveness. Adolescence is characterized by increased emotional sensitivity and ongoing maturation of cognitive control systems (Pine et al., 1998), suggesting that regulatory strategies effective in adults may not function identically in younger populations. A systematic evaluation of adolescent-specific evidence is therefore needed to clarify both effectiveness and contextual relevance.

The present study systematically reviews controlled and quasi-experimental studies investigating the effects of music-based interventions on anxiety among adolescents. In addition to examining intervention outcomes, the review considers how different formats—such as passive listening, structured music therapy, and active music-making—may engage distinct yet overlapping regulatory processes. Prior neuroscientific and psychophysiological research indicates that music can influence emotional appraisal systems (Koelsch, 2014), modulate autonomic and endocrine stress responses (Bernardi et al., 2006; Chanda and Levitin, 2013), and facilitate social bonding and self-expression (Rickson and Watkins, 2003). However, these mechanisms are rarely discussed in relation to adolescent intervention studies in a unified manner. By examining patterns across studies, the present review seeks to identify recurring processes that may help explain observed anxiety reductions.

By bringing together and critically appraising this diverse body of evidence, the review aims to clarify the extent to which music-based approaches can alleviate anxiety in adolescents and to identify methodological and contextual factors that influence outcomes. Particular attention is given to intervention setting (e.g., school versus clinical environments), participant characteristics, and measurement approaches, as these variables may account for inconsistencies in reported effects. Through this synthesis, the review contributes to a more developmentally grounded understanding of how music may function as a regulatory resource during adolescence and highlights directions for future research that integrates psychological, physiological, and contextual dimensions of anxiety reduction.

## 2 Materials and methods

### 2.1 Protocol and registration

This systematic review followed the Preferred Reporting Items for Systematic Reviews and Meta-Analyses (PRISMA 2020) guidelines to ensure methodological transparency and rigor (Page et al., 2021). The review protocol was prospectively registered in the International Prospective Register of Systematic Reviews (PROSPERO, registration ID: CRD420251129625), which provides an *a priori* specification of the review methods and helps reduce reporting bias and unplanned deviations from the protocol.

### 2.2 Search strategy

This systematic review search strategy was developed to comprehensively identify studies examining the effects of music-related interventions on anxiety and related emotional outcomes among adolescents. A comprehensive electronic search was carried out across five major databases: PubMed, APA PsycINFO (American Psychological Association PsycINFO database), Scopus, Web of Science, and CNKI (China National Knowledge Infrastructure). The search covered publications from the earliest available year in each database up to June 6, 2025. No language restrictions were initially applied; however, only articles published in English or Chinese were retained for full-text screening to ensure the feasibility of analysis. The search strategy integrated three key conceptual domains: intervention (music-related approaches), psychological outcome (anxiety and emotional distress), and population (adolescents). For each domain, a set of synonyms and related terms was developed to maximize search sensitivity. The terms were combined using Boolean operators (“AND”/ “OR”) to refine the search scope. Table 1 shows the search terms in the database.

**Table 1:** Search terms in the database.

Search terms	
Intervention	“music intervention” OR “music therapy” OR “music listening” OR “background music” OR “music exposure”
Outcome	“anxiety” OR “stress” OR “nervousness” OR “emotional distress” OR “anxiety disorders” OR “psychological distress” OR “emotional state”
Population	“adolescents” OR “teenagers” OR “youth” OR “young people” OR “secondary school students”

The complete search string applied across databases was structured as follows: (“music intervention” OR “music therapy” OR “music listening” OR “background music” OR “music exposure”) AND (“anxiety” OR “stress” OR “nervousness” OR “emotional distress” OR “anxiety disorders” OR “psychological distress” OR “emotional state”) AND (“adolescents” OR “teenagers” OR “youth” OR “young people” OR “secondary school students”).

To align with database-specific indexing systems and search functionalities, minor adjustments were made to the search string while preserving the core logic: In Scopus, the search was restricted to title, abstract, and keyword fields using the “TITLE-ABS-KEY” tag. In APA PsycINFO, terms were searched across all fields using the “Any Field” parameter to ensure broad coverage. In CNKI, the search was adapted to Chinese-language indexing conventions while retaining the conceptual consistency of the original terms. In addition to database searching, reference lists of relevant reviews and included studies were manually screened, and Google Scholar was consulted to capture potential grey literature. The first 200 records sorted by relevance were screened, in line with common systematic review practice. These steps ensured broader coverage and minimized the risk of publication bias. Table 2 presents the comprehensive search strategy for all databases.

**Table 2:** The total number of hits for the entire search strategy for the databases.

Databases	Complete search strategy	Results
PubMed (1995–2025)	((“music intervention” OR “music therapy” OR “music listening” OR “background music” OR “music exposure”) AND (“anxiety” OR “stress” OR “nervousness” OR “emotional distress” OR “anxiety disorders” OR “psychological distress” OR “emotional state”)) AND (“adolescents” OR “teenagers” OR “youth” OR “young people” OR “secondary school students”)	85
APA PsycINFO (1986–2025)	Any Field: “music intervention” OR “music therapy” OR “music listening” OR “background music” OR “music exposure” AND Any Field: “anxiety” OR Any Field: “stress” OR Any Field: “nervousness” OR Any Field: “emotional distress” OR Any Field: “anxiety disorders” OR Any Field: “psychological distress” OR Any Field: “emotional state” AND Any Field: “adolescents” OR Any Field: “teenagers” OR Any Field: “youth” OR Any Field: “young people” OR Any Field: “secondary school students”	130
Scopus (1975–2025)	(TITLE-ABS-KEY (“music intervention” OR “music therapy” OR “music listening” OR “background music” OR “music exposure”) AND TITLE-ABS-KEY (“anxiety” OR “stress” OR “nervousness” OR “emotional distress” OR “anxiety disorders” OR “psychological distress” OR “emotional state”) AND TITLE-ABS-KEY (“adolescents” OR “teenagers” OR “youth” OR “young people” OR “secondary school students”))	451
Web of Science (1995–2025)	“music intervention” OR “music therapy” OR “music listening” OR “background music” OR “music exposure” (All Fields) AND “anxiety” OR “stress” OR “nervousness” OR “emotional distress” OR “anxiety disorders” OR “psychological distress” OR “emotional state” (All Fields) AND “adolescents” OR “teenagers” OR “youth” OR “young people” OR “secondary school students” (All Fields)	211
CNKI (1985–2025)	TITLE-ABS-KEY (“music intervention” OR “music therapy” OR “music listening” OR “background music” OR “music exposure”) AND TITLE-ABS-KEY (“anxiety” OR “stress” OR “nervousness” OR “emotional distress” OR “anxiety disorders” OR “psychological distress” OR “emotional state”) AND TITLE-ABS-KEY (“adolescents” OR “teenagers” OR “youth” OR “young people” OR “secondary school students”)	85

### 2.3 Eligibility criteria

In this review, the PICOS framework was applied to guide the selection of eligible studies. Adolescence was defined according to the World Health Organization as individuals aged 10–19 years. PICOS represents five elements: (1) Population, (2) Intervention, (3) Comparison, (4) Outcome, and (5) Study design. These components served as the inclusion criteria when screening the literature. Only those studies that fulfilled all relevant PICOS requirements were included in the analysis. The detailed inclusion and exclusion criteria are presented in Table 3.

**Table 3:** PICOS-based inclusion criteria.

Items	Detailed inclusion criteria
Population	Adolescents (10–19 years; WHO definition), with or without existing mental health concerns
Intervention	Any form of music-based intervention
Comparison	Any different intervention or no intervention
Outcome	Measures of anxiety or emotional states that included anxiety-related indicators
Study designs	Randomized controlled trials (RCTs) or Quasi-experimental

Studies were excluded if they met any of the following conditions: (1) participants were not adolescents; (2) the intervention did not involve a music-based component; (3) anxiety was not assessed as a primary or secondary outcome; (4) the design was not a randomized controlled trial or quasi-experimental study; (5) the publication was non-empirical (e.g., review, editorial, commentary); or (6) insufficient methodological details were provided. Eligibility decisions were made in accordance with the predefined PICOS criteria specified in the registered protocol.

### 2.4 Study selection

**Figure 1:**
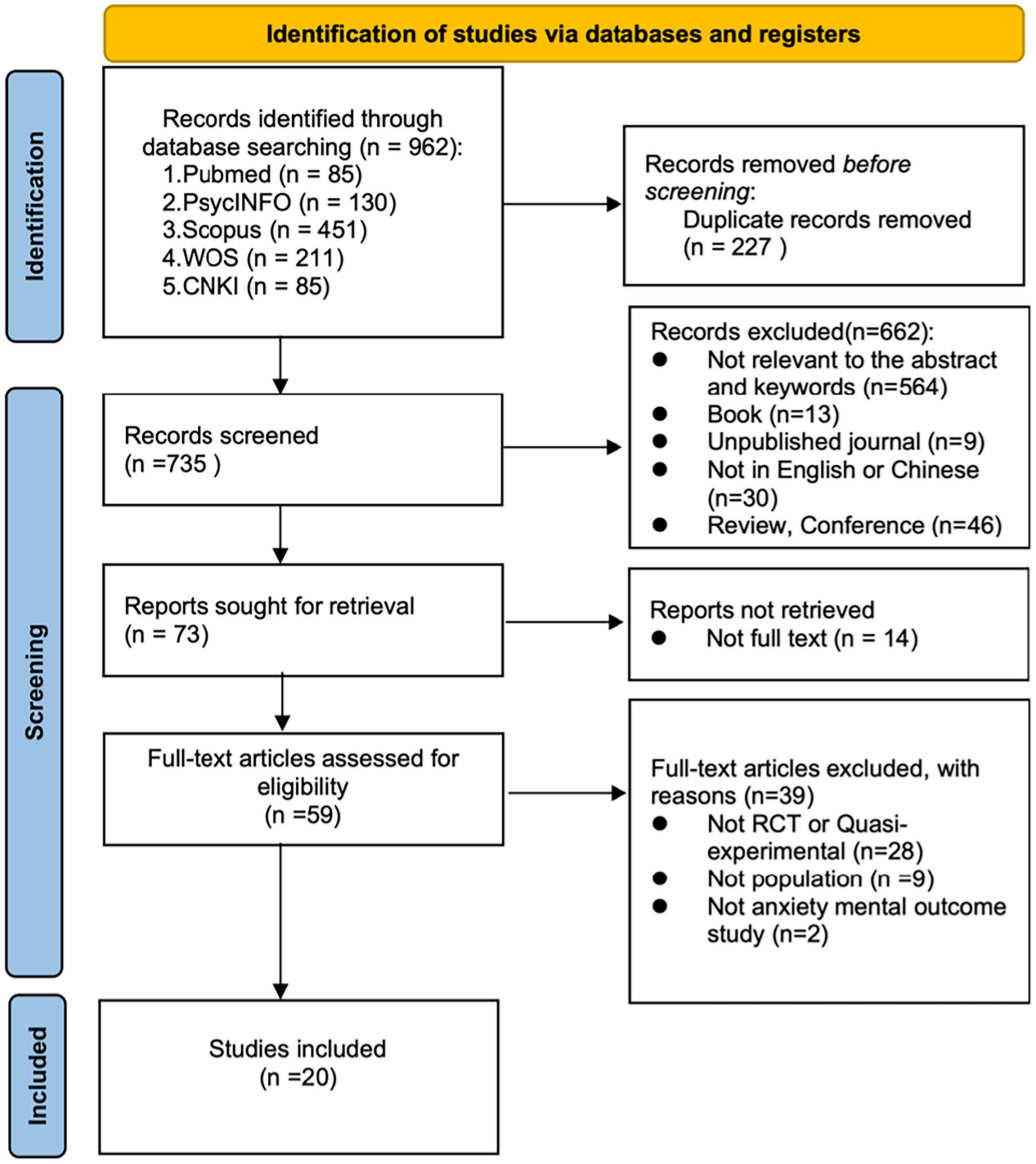
Preferred reporting items for systematic reviews and meta-analysis (PRISMA 2020) flow diagram of the study screening process.

Study selection proceeded in three stages: (1) title and abstract screening, (2) full-text review, and (3) final inclusion. All records were imported into EndNote 20, and duplicate entries were removed automatically and manually. Two independent reviewers screened titles and abstracts against the eligibility criteria. Full texts of potentially relevant studies were then independently assessed. Discrepancies between reviewers were resolved through discussion until consensus was reached. The study selection process is illustrated in the PRISMA 2020 flowchart (Figure 1).

### 2.5 Data extraction and quality assessment

After completing the literature search, data from all eligible studies were systematically extracted using a standardized form. The extracted information included: (1) bibliographic details such as author, title, country and publication year; (2) sample size and presence of control group; (3) demographic characteristics of participants (e.g., age, gender); (4) intervention details, including type, duration, and frequency; (5) study design; and (6) main findings related to anxiety outcomes. Data extraction was carried out independently by one reviewer and subsequently verified by a second reviewer to ensure accuracy and consistency.

The methodological quality of the included studies was evaluated using the Physiotherapy Evidence Database (PEDro) scale,^1^ a tool designed to evaluate the methodological quality of experimental designs. It has proven to be valuable when developing systematic reviews due to its high validity and reliability (Moseley et al., 2015). The PEDro scale consists of 11 items that examine critical aspects of trial quality, including randomization procedures, allocation concealment, blinding of participants, therapists, and assessors, adequacy of follow-up, intention-to-treat analysis, between-group comparisons, and reporting of statistical estimates with variability.^2^ Each item is scored as either “Yes” (1 point) or “No” (0 points), except for the first item on eligibility criteria, which addresses external validity and is not included in the total score, indicated by “Y” (yes) or “N” (no).

Two independent evaluators with relevant methodological training performed the quality assessment. Any disagreements were discussed and resolved through consensus. The final PEDro scores range from 0 to 10, with higher scores indicating stronger methodological quality (Maher et al., 2003). In this review, studies scoring 8–10 were classified as high quality, scores of 5–7 as moderate to good quality, scores of 3–4 as fair, and scores below 3 as poor (Albanese et al., 2020). Blinding criteria were applied conservatively, particularly for participant and therapist blinding, which are typically not feasible in music-based interventions. This systematic evaluation provided a structured basis for interpreting the reliability of the included evidence.

### 2.6 Data synthesis

Due to substantial heterogeneity across the included studies in terms of intervention type, participant characteristics, study settings, outcome measures, intervention duration, and reporting methods, a meta-analysis was not considered appropriate. Instead, findings were synthesized narratively following the principles of the Synthesis Without Meta-analysis (SWiM) reporting guideline (Campbell et al., 2020).

Studies were first grouped according to intervention type, including receptive music listening, active music participation, music therapy programs, and mixed music-based interventions. Findings were subsequently compared across different settings (e.g., schools, hospitals, and community environments), participant populations, outcome domains, intervention duration, and outcome-assessment timing.

The synthesis focused on identifying patterns of consistency and variation in anxiety-related outcomes across studies. Particular attention was given to differences in intervention characteristics, participant contexts, and follow-up periods. A quantitative meta-analysis was not conducted because the included studies employed diverse anxiety measures, intervention protocols, outcome reporting formats, and follow-up schedules, limiting statistical comparability across studies. In addition, quantitative effect estimates, mean differences, and 95% confidence intervals were not reported consistently across the included studies. Consequently, standardized quantitative synthesis of effect sizes was not feasible, and findings were synthesized narratively with emphasis placed on the direction, consistency, and overall pattern of reported intervention effects.

## 3 Results

### 3.1 Study selection

Figure 1 illustrates a systematic search of databases (85 from PubMed, 130 from APA PsycINFO, 451 from Scopus, 211 from Web of Science, and 85 from CNKI), which identified 962 initial records. After removing 227 duplicates, 735 records remained for screening. During title-and-abstract screening, 662 records were excluded, including 564 non-relevant records, 13 books, 9 unpublished journals, 30 non-English/Chinese publications, and 46 reviews or conference papers. Seventy-three reports were sought for retrieval, but 14 were not retrieved due to a lack of full text. For the 59 full-text articles assessed for eligibility, 39 were excluded: 28 were not randomized or quasi-experimental studies, 9 did not target the relevant population, and 2 were not mental health outcome studies. Ultimately, 20 studies met all inclusion criteria and were included in the systematic review.

### 3.2 Study quality assessment

Table 4 presents the PEDro quality ratings for all 20 included studies. Several methodological domains were consistently well met. All studies clearly specified their eligibility criteria, and all achieved adequate follow-up (>85%), indicating strong retention across interventions. Likewise, every study reported appropriate between-group comparisons and provided point estimates with measures of variability, demonstrating solid and uniform reporting in these core areas.

**Table 4:** Summary of methodological quality assessment scores.

**References**	**Eligibility criteria (Y/N)**	**Random allocation (0/1)**	**Concealed allocation (0/1)**	**Baseline comparability (0/1)**	**Blind subjects (0/1)**	**Blind therapists (0/1)**	**Blind assessors (0/1)**	**Adequate Follow-up (0/1)**	**Intention to Treat Analysis (0/1)**	**Between group comparisons (0/1)**	**Point estimates and variability (0/1)**	**PEDro** **Score (0–10)**
Iyendo et al. (2024)	Y	1	0	1	0	0	0	1	0	1	1	5
Porter et al. (2017)	Y	1	1	1	0	0	1	1	1	1	1	8
Kim et al. (2024)	Y	1	1	1	0	0	1	1	1	1	1	8
Saghaee-Shahriari and Mostafazadeh (2019)	Y	0	0	1	0	0	0	1	0	1	1	4
Bong et al. (2021)	Y	1	0	0	0	0	0	1	0	1	1	4
Park et al. (2023)	Y	1	0	1	0	0	1	1	0	1	1	6
Zhou et al. (2025)	Y	0	0	1	0	0	0	1	0	1	1	4
Yi et al. (2012)	Y	1	0	1	0	0	0	1	0	1	1	5
Uhlig et al. (2018)	Y	1	1	1	0	0	1	1	1	1	1	8
Ugwuanyi et al. (2020)	Y	1	1	1	0	0	1	1	1	1	1	8
Scheufler et al. (2021)	Y	1	1	1	0	0	1	1	1	1	1	8
Rahmani et al. (2016)	Y	1	0	1	0	0	0	1	1	1	1	6
Pérez-Eizaguirre et al. (2022)	Y	0	0	1	0	0	0	1	1	1	1	5
Nelson et al. (2017)	Y	1	1	1	0	0	1	1	0	1	1	7
Matokhniuk et al. (2021)	Y	0	0	1	0	0	0	1	1	1	1	5
Kwok (2019)	Y	1	1	1	0	0	1	1	1	1	1	8
Colwell et al. (2013)	Y	0	0	1	0	0	1	1	0	1	1	5
Chen et al. (2022)	Y	1	1	1	0	0	1	1	1	1	1	8
Chen et al. (2019)	Y	1	1	1	0	0	1	1	1	1	1	8
Alemdar et al. (2023)	Y	1	1	1	0	0	1	1	1	1	1	8

Greater variability emerged in other methodological items. Random allocation was described in most trials but was unclear in several studies, including Iyendo et al. (2024), Rahmani et al. (2016), Matokhniuk et al. (2021), and Park et al. (2023). In several of these studies, baseline comparability between groups suggested that group allocation was likely balanced, although the absence of explicit reporting limits certainty. Allocation concealment was the least frequently satisfied criterion; only a subset of randomized trials reported concealment procedures, including Porter et al. (2017), Kim et al. (2024), Ugwuanyi et al. (2020), Uhlig et al. (2018), Scheufler et al. (2021), Kwok (2019), Chen et al. (2019), and Chen et al. (2022). As expected for music-based interventions, no study achieved participant or therapist blinding. This reflects an inherent methodological constraint rather than a deficiency in study design, as such interventions require active engagement or perceptual awareness of music. Therefore, blinding of participants and therapists is generally not feasible in this context and should not be interpreted as indicative of poor methodological quality. In contrast, assessor blinding—such as outcome evaluation or statistical analysis—was implemented in a subset of higher-quality trials.

Based on total PEDro scores, no study achieved a maximum score of 10. The highest quality rating observed was 8/10, achieved by several studies, including Ugwuanyi et al. (2020), Alemdar et al. (2023), Porter et al. (2017), Kim et al. (2024), Uhlig et al. (2018), Scheufler et al. (2021), Kwok (2019), Chen et al. (2019), and Chen et al. (2022). The remaining studies fell within the moderate range (5–7 points)—for example, Nelson et al. (2017), Iyendo et al. (2024), Rahmani et al. (2016), Pérez-Eizaguirre et al. (2022), Yi et al. (2012), Colwell et al. (2013), Park et al. (2023), and Matokhniuk et al. (2021)—typically due to unclear randomization processes, lack of allocation concealment, or absence of assessor blinding. The lowest scores (4/10, corresponding to fair methodological quality) were observed for Saghaee-Shahriari and Mostafazadeh (2019), Bong et al. (2021), and Zhou et al. (2025), where randomization, blinding, and reporting details were limited.

Overall, while blinding and allocation concealment were inconsistently applied, the strong consistency in eligibility reporting, follow-up, between-group comparisons, and statistical estimates supports the overall interpretability of findings across studies. These methodological patterns should be considered when assessing the robustness of the overall evidence.

### 3.3 Population characteristics

The population characteristics of the 20 studies included in this review are summarized below: (1) Sample size: Altogether, the 20 studies involved 1,912 participants, with sample sizes ranging from 22 to 470. This variation reflects differences in study design and feasibility across settings. The variation in sample sizes likely reflects differences in primary outcomes, expected effect sizes, intervention contexts, and study objectives, all of which influence sample size requirements across studies. (2) Gender: 18 studies recruited both male and female participants, while one study did not report gender distribution, and one focused exclusively on female adolescents. This uneven reporting highlights a potential limitation in understanding gender-specific effects. (3) Age: All studies primarily targeted adolescent populations. Within these, three studies included younger children alongside adolescents, and one extended the age range up to 21 years. Nevertheless, adolescents (aged roughly 12–18 years) remained the central focus across all studies, ensuring that the evidence base is highly relevant to this developmental stage. (4) Health status: Considerable heterogeneity was observed in participant health conditions. Specifically, 10 studies recruited adolescents with psychological problems (e.g., anxiety, stress, or depressive symptoms), and four studies involved participants with physical health issues (such as chronic illness or medical conditions). In comparison, 6 studies targeted healthy school-based adolescents. This distribution suggests that while clinical and at-risk populations have been extensively investigated, a substantial body of evidence remains focused on preventive and non-clinical contexts.

In summary, the studies reviewed encompassed a diverse range of adolescent populations, varying in terms of sample size, gender composition, age distribution, and health status. While many studies emphasized adolescents with psychological or physical problems, a notable portion also examined healthy school-based participants, thereby offering insights into both clinical and preventive contexts. This diversity strengthens the evidence base but also underscores the need for more balanced investigations across different adolescent groups. The detailed population characteristics are presented in Table 5.

**Table 5:** Key characteristics of the 20 studies included in the review.

**Study**	**Country**	**Population**			**Intervention**				**Outcome**		
		**Sample**	**Age**	**Gender**	**Study design**	**Type**	**Duration**	**Frequency**	**Measures**	**Key findings**	**Outcome assessment/Follow-up timing**
Iyendo et al. (2024)	Nigeria	*N* = 470 Adolescents with PTSD	13–18	Mixed	RCT	Audio-visual-based art and music listening	6 weeks	5 times/week	International Trauma Questionnaire (ITQ)	PTSD symptoms ↓ Emotional regulation ↑ Attention ↑	Immediately post-intervention assessment
Porter et al. (2017)	Northern Ireland	*N* = 251 (111M, 140F) Children and adolescents with emotional and behavioral difficulties	8–16	Mixed	RCT	The Alvin model of “Free Improvisation” involves creating sounds and improvising	12 weeks	1 time/week	SSIS; Rosenberg Self-Esteem Scale; CES-DC; Child Behavior Checklist;	Improved prosocial behavior ↑ Decreased behavioral problems ↓ Depression levels ↓	13-week post-intervention assessment and 26-week follow-up
Kim et al. (2024)	South Korea	*N* = 50 Adolescents	14–17	Mixed	RCT	Wind instrument (flute) performance and choir training	12 weeks	2 times/week	PWI-SF; Pulmonary function test; Respiratory muscle pressure test; WHOQOL-BREF	Respiratory function ↑ Anxiety level ↓ Life well-being ↑	Immediately post-intervention assessment
Saghaee-Shahriari and Mostafazadeh (2019)	Iran	*N* = 30 Adolescents with leukemia	13–18	Mixed	Quasi-experimental	Receptive music therapy (music listening)	7 weeks	2 times/week	Anxiety Sensitivity Index (ASI); General Self-Efficacy Scale (GSE)	Anxiety sensitivity ↓ Self-efficacy ↑	Immediately post-intervention assessment
Bong et al. (2021)	South Korea	*N* = 155 Adolescents with smartphone/internet addiction	10–16	Mixed	RCT	Composing, singing and playing musical instruments	8 weeks	1 time/week	YIAT; SAPS; SAIC/TAIC; CASS(S); BDI-11; BIS-11; RSES	Internet addiction ↓ Anxiety level ↓ Impulsiveness ↓	Immediately post-intervention assessment
Park et al. (2023)	South Korea	*N* = 36 Adolescents and children with ADHD	7–15	Mixed	RCT	Improvisation and music listening	12 weeks	2 times/week	Neurophysiological indicators; CDI; DHQ	Physiological stress level ↓ Psychological anxiety level ↓	Immediately post-intervention assessment
Zhou et al. (2025)	China	*N* = 120 (69M, 51F) Adolescents with depression	13–18	Mixed	Quasi-experimental	Family music creation; Music appreciation and sharing; Music meditation and relaxation; Music activities and games	12–16 weeks	1 time/week	HAMD; HAMA; FAD; ASLEC; PSQI	Depression level ↓ Anxiety level ↓ Sleep quality ↑ Self-life happiness identity ↑	Immediate post-intervention assessment
Yi et al. (2012)	China	*N* = 70 (25M, 45F) Adolescents with emotional disorders	12–18	Mixed	RCT	Percussion rhythm training; Listening to music; Lyrics filling; Music painting	3 weeks	2 times/week	HAMA; HAMD	Depression level ↓ Anxiety level ↓ Emotional communication ↑	Immediately post-intervention assessment
Uhlig et al. (2018)	Netherlands	*N* = 90 (40M, 55F) Adolescents	8–13	Mixed	RCT	Rhythmic engagement; Singing and rapping; Lyrics writing and filling	16 weeks	1 time/week	SDQ; DERS; SPPC; CBSK	Negative emotions (anxiety, anger) ↓; Self-esteem ↑; Emotional regulation ability ↑;	Baseline and post-intervention assessment (4 months)
Ugwuanyi et al. (2020)	Nigeria	*N* = 83 (46M, 37F) Adolescents	13–18	Mixed	RCT	Critical listening to the musical material; Songwriting; Playing musical instruments	12 weeks	1 time/week	Generalized test anxiety inventory	Physics test anxiety level ↓	Immediately post-intervention assessment
Scheufler et al. (2021)	USA	*N* = 48 (8M, 40F) Adolescents with APS	10–18	Mixed	RCT	LPSM: Song singing and music movement use 25 songs from mu- sisal genres including pop, rock, country, alternative, and oldies; AME: Chant writing (fill-in-the-blank method), instrument improvisation; MAR: Music Listening and Imagining	3 weeks	1 time/week	STICSA-C; VAS	Anxiety level ↓; Relaxation level ↑	Immediately post-intervention assessment
Rahmani et al. (2016)	Iran	*N* = 24 Adolescents with depressive symptoms	15	Only Female	RCT	Art therapy: Painting with music listening; Music therapy: Music listening with three music themes exhilarating, sad, and strengthening.	7 weeks	1 time/week	BDI; CDI	Depression level ↓; Positive emotions ↑	Immediately post-intervention assessment
Pérez-Eizaguirre et al. (2022)	Spain	*N* = 22 (12M, 10F) Adolescents committed CPV	13–21	Mixed	Quasi-experimental	Creative improvisation; Song singing; Rhythm Interaction	8 weeks	1 time/week	State–Trait Anxiety Inventory (STAI)	State and trait anxiety levels ↓	Immediately post-intervention assessment
Nelson et al. (2017)	USA	*N* = 44 Adolescents with idiopathic scoliosis	10–19	Mixed	RCT	Watching music videos and relaxation training before surgery; Music listening and imagination after surgery	1 week	2 times/week	A 0–10 numeric rating scale (NRS)	Anxiety level ↓ Pain level ↓	Pre-operative and post-operative assessment
Matokhniuk et al. (2021)	Ukraine	*N* = 60 Adolescents	13–15	Not specified	Quasi-experimental	Singing; Playing musical instruments and rhythmic recitation; Motor dramatization to music; Musical story	12 weeks	2 times/week	Spielberger-J. Khanin Anxiety Scale; Phillips’ School Anxiety Scale; Eisenko’s Personality Questionnaire; Ilyin’s Shyness and Timidity Scale	Anxiety level ↓ Emotional control ability ↑ Shyness level ↑ Self-esteem and self-confidence ↑	Immediately post-intervention assessment
Kwok (2019)	China	*N* = 106 (74M, 32F) Adolescents	13–15	Mixed	RCT	Music listening; Playing musical instruments; Follow the music and imagine; Role-playing with music	8 weeks	1 time/week	CHS; WLEIS; HADS; SHS	Anxiety level ↓ Emotional Competence ↑ Subjective happiness ↑ Hope ↑	Immediately post-intervention assessment
Colwell et al. (2013)	USA	*N* = 32 (15M, 17F) Hospitalized children and adolescents	6–17	Mixed	Quasi-experimental	Music listening; Music composition; Orff-based therapy	1 week	1 time/week	Wong–Baker FACES Pain Rating Scale; STAIC	Anxiety level ↓ Pain level ↓	Immediately post-intervention assessment
Chen et al. (2022)	China Taiwan	*N* = 56 Adolescents with parental attachment insecurity	10–19	Mixed	RCT	A group music-based intervention (Warm up with light music, music listening and karaoke singing)	10 weeks	2 times/week	IPPA; C-YSR	Parental attachment insecurity ↓ Anxiety level ↓	Immediately post-intervention assessment
Chen et al. (2019)	China Taiwan	*N* = 66 Adolescents	12–13	Mixed	RCT	Music warm-up, preferred music listening, karaoke singing, classical music relaxation, instrument interaction	10 weeks	2 times/week	IPA; BDI-II; Salivary cortisol concentrations	Peer attachment ↓ Depression and anxiety levels ↓ Salivary cortisol levels ↓	Immediately post-intervention assessment
Alemdar et al. (2023)	Turkey	*N* = 99 Adolescents treated in the pediatric intensive care unit	12–18	Mixed	RCT	Listening to classical music (Choose slow classical music with a tempo of 60 beats per minute no strong chords or minor keys, mainly in major keys)	1 week	1 time/week	WB-FACES; CFS	Anxiety level ↓ Pain level ↓ Fear level ↓	Immediately post-intervention assessment

Outcome assessment timing was predominantly immediate post-intervention across the included studies. Only a limited number of studies reported assessments beyond the immediate post-intervention period, including Porter et al. (2017), which included 13-week and 26-week follow-up assessments. Where explicit follow-up information was not reported, outcome assessment timing was classified based on the timing of data collection described in the original study. *PTSD: Post-traumatic stress disorder; SSIS: Social Skills Improvement System Rating Scales; CES-DC: Centre for Epidemiological Studies Depression Scale for Children; PWI-SF: Psychological social well-being index short form; WHOQOL-BREF: World Health Organization Quality of Life Scale Abbreviated Version; YIAT: Young’s Internet Addiction Test; SAPS: Smartphone Addiction Proneness Scale; SAIC/TAIC: State/Trait Anxiety Inventory for Children; CASS(S): Conners-Wells’ Adolescent Self-Report Scale Short Form; BDI-11: Beck Depression Inventory-II; BIS-11: Barratt Impulsiveness Scale-11; RSES: Rosenberg Self Esteem Scale; ADHD: attention-deficit hyperactivity disorder; CDI: Children’s depression inventory; DHQ: Daily hassles questionnaire; HAMD: Hamilton Depression Rating Scale; HAMA: Hamilton Anxiety Rating Scale; FAD: Family Assessment Device; ASLEC: Adolescent Self-Rating Life Events Checklist; PSQI: Pittsburgh Sleep Quality Index; SDQ: Strength and Difficulty Questionnaire; DERS: Difficulties in Emotion Regulation Scale; SPPC: Self-perception Profile for Children; CBSK: Competentie Belevingss- chaal voor kinderen (Dutch version); APS: Amplified pain syndrome; LPSM: Live patient-selected music; AME: Active music engagement; MAR: Music-assisted relaxation; STICSA-C: State–Trait Inventory for Cognitive and Somatic Anxiety for Children; VAS: Visual Analog Scale; BDI: Beck Depression Inventory; CDI: Children’s Depression Inventory; CPV: Child to Parent Violence; CHS: Children’s Hope Scale (Chinese version); WLEIS: Wong and Law Emotional Competence Scale; HADS: Hospital Anxiety and Depression Scale (Chinese version); SHS: Subjective Happiness Scale (Chinese version); IPPA: Inventory of parent and peer attachment (the paternal attachment, maternal attachment, and peer attachment subscales); C-YSR: Chinese version of the youth self-report.; IPA: The Inventory of Peer Attachment; WB-FACES: Wong–Baker FACES Pain Rating Scale; CFS: Children’s Fear Scale. Higher ↑; Lower ↓.

### 3.4 Intervention characteristics

Table 5 summarizes the main intervention features across the 20 included studies, covering intervention type, duration, and frequency. In terms of intervention type, all studies employed music-based interventions, though the specific formats varied. Some focused on active participation, such as group singing, instrument playing, or structured music therapy sessions, while others employed passive listening approaches, typically involving exposure to background or recorded music. A few studies have combined music interventions with complementary strategies, such as relaxation exercises or cognitive training, reflecting a growing interest in multimodal approaches.

Regarding intervention duration, considerable variation was observed across the studies. The shortest program consisted of a single one-off intervention delivered within 1 week, while the longest extended to 16 weeks. Most studies, however, implemented interventions lasting between 6 and 12 weeks, suggesting that this timeframe is both practical in school and clinical contexts and sufficient to detect meaningful changes in anxiety outcomes.

As for intervention frequency, most studies conducted sessions between one and three times per week. Several trials reported higher frequencies, such as daily exposure in school-based settings, whereas a small number did not clearly specify the session schedule. Session length also varied, ranging from 20 min to over 1 h; however, most interventions were delivered in formats of approximately 30–45 min per session, which appears to strike a balance between feasibility and therapeutic benefit.

### 3.5 Outcome

This review synthesized findings from 20 studies, all of which assessed anxiety as a primary or secondary outcome. While the core emphasis was on anxiety, several investigations also considered additional psychological, behavioral, or physiological outcomes, thereby offering a broader view of how music interventions affect adolescents.

#### 3.5.1 Anxiety as the primary outcome

Across the 20 studies, reductions in anxiety were consistently reported, although the magnitude of the effects varied according to the intervention type, study design, and context.

School-based interventions: Several studies focusing on non-clinical adolescent populations reported significant reductions in anxiety. For instance, Ugwuanyi et al. (2020) showed that exposure to music-based activities, including listening, singing, and instrumental play, alleviated examination-related anxiety. Similarly, Kwok (2019) demonstrated that structured music sessions enhanced emotional competence and subjective well-being while reducing anxiety symptoms. Uhlig et al. (2018) found that rhythmic and improvisational activities lowered anxiety and anger while improving self-esteem and emotional regulation.

Clinical and medical settings: In studies involving adolescents undergoing medical procedures, music interventions have been shown to reduce situational anxiety effectively. Nelson et al. (2017) reported that music video sessions, alongside relaxation training, decreased both anxiety and pain in post-surgical contexts. Alemdar et al. (2023) found that listening to classical music significantly reduced anxiety, fear, and pain among pediatric patients.

Group and family contexts: Chen et al. (2022) revealed that group-based music interventions, combining listening, karaoke, and social interaction, reduced adolescent anxiety while improving attachment security. Porter et al. (2017) reported that improvisational music therapy reduced depressive symptoms and behavioral problems, with improvements in prosocial behaviors indirectly reflecting lowered anxiety.

Taken together, these results indicate that both active engagement (e.g., playing, singing, or improvisation) and passive listening can mitigate anxiety, although active approaches generally produced stronger and longer-lasting benefits.

#### 3.5.2 Secondary emotional and psychological outcomes

In addition to anxiety, several studies assessed broader emotional or psychological outcomes, many of which showed concurrent improvements.

Depression and stress: Porter et al. (2017) reported reductions in depression and behavioral difficulties, while Rahmani et al. (2016) documented decreased depression and improved positive affect following combined art and music listening sessions. Zhou et al. (2025) found that family-based music-making and music meditation not only lowered anxiety but also reduced depressive symptoms and improved sleep quality. Yi et al. (2012) showed that percussion-based rhythm training accompanied by music listening led to declines in both anxiety and depression.

Emotional regulation and well-being: Uhlig et al. (2018) and Kwok (2019) highlighted improved emotional regulation and enhanced well-being in adolescents following music-based interventions. Iyendo et al. (2024) demonstrated that a combined visual art and music program enhanced emotion regulation and reduced PTSD symptoms. Park et al. (2023) reported simultaneous reductions in psychological anxiety and physiological stress, while Saghaee-Shahriari and Mostafazadeh (2019) observed decreases in anxiety sensitivity alongside improvements in self-efficacy.

Gender- and subgroup-specific findings were reported inconsistently across the included studies. Porter et al. (2017) observed that some psychosocial outcomes appeared to improve more substantially among female participants, suggesting that gender may influence responsiveness to music-based interventions. However, most studies did not conduct formal gender-stratified analyses, and gender-specific findings were rarely reported. Studies such as Uhlig et al. (2018) and Kwok (2019) demonstrated positive psychosocial outcomes in mixed-gender samples but did not provide sufficient subgroup data to determine whether intervention effects differed by gender. Therefore, although preliminary evidence suggests that gender may play a role in psychosocial responses to music interventions, current findings remain insufficient to draw definitive conclusions regarding gender-specific effects among adolescents.

These findings suggest that music interventions often yield multidimensional psychological benefits, whereby broader improvements in emotional functioning, well-being, and psychosocial adjustment may accompany reductions in anxiety.

#### 3.5.3 Physiological and behavioral correlates

A smaller group of studies included physiological or behavioral measures that corroborated psychological outcomes.

Physiological indicators: Chen et al. (2019) reported that music therapy reduced salivary cortisol, aligning with self-reported reductions in anxiety and depression. Park et al. (2023) documented improvements in neurophysiological markers alongside reduced anxiety. Kim et al. (2024) showed that wind instrument training enhanced respiratory function while reducing anxiety, suggesting a link between rhythmic breathing and emotional regulation.

Behavioral and social outcomes: In addition to symptom reduction, several studies recorded behavioral gains. Porter et al. (2017) found that adolescents engaged in improvisational music therapy displayed increased prosocial behavior and fewer behavioral problems. Similarly, Chen et al. (2022) noted improved attachment-related outcomes, indicating that reductions in anxiety may enhance interpersonal functioning.

#### 3.5.4 Intervention characteristics and dose–response patterns

Intervention modality: Both passive listening and active engagement have been shown to be effective. For example, studies using passive music listening reported positive outcomes (Alemdar et al., 2023; Nelson et al., 2017), while those incorporating active participation—such as singing, playing instruments, or improvisation—tended to produce more substantial and sustained reductions in anxiety (Chen et al., 2022; Ugwuanyi et al., 2020; Uhlig et al., 2018).

Duration and frequency: Most interventions lasted 8–12 weeks with weekly or bi-weekly sessions. However, short-term approaches also proved effective: Colwell et al. (2013) demonstrated reductions in anxiety and pain after just a single week of music-based relaxation. Conversely, Zhou et al. (2025) reported that longer interventions often showed broader and more lasting benefits, including improved sleep and overall well-being.

Context of delivery: School-based interventions were especially effective for preventive purposes, whereas hospital-based interventions often focused on acute anxiety reduction in high-stress situations, such as surgery or medical treatment.

Overall, across the 20 studies, the evidence demonstrates that music interventions consistently reduce anxiety among adolescents, with active participation generally producing more substantial and enduring effects than passive listening. Improvements in anxiety often co-occur with reductions in depression and stress, enhanced emotional regulation, and, in some cases, favorable changes in physiological indicators. Where subgroup analyses were conducted, gender differences in response to music interventions were not consistently reported. Although some studies suggested the possibility of differential psychosocial responses, the available evidence remains insufficient to determine whether music interventions exert stronger effects in either gender.

Follow-up duration varied considerably across studies, with most assessing outcomes immediately following intervention completion and only a limited number incorporating follow-up assessments beyond the intervention period, limiting conclusions regarding sustained effects. School-based programs highlight the preventive potential of music, while medical and family contexts underscore its applicability in high-stress and therapeutic settings.

## 4 Discussion

This systematic review synthesized evidence from 20 studies investigating how music-based interventions influence anxiety in adolescents. Across the reviewed literature, music listening and music-related activities were consistently associated with reduced anxiety symptoms. Importantly, these findings suggest that music functions not merely as a relaxation tool, but as a psychologically meaningful stimulus capable of engaging emotional and attentional processes during a critical developmental period, which is consistent with previous research highlighting the role of music in emotion regulation and neural processing (Koelsch, 2014; Thoma et al., 2013).

### 4.1 Active vs. receptive music interventions

Several studies highlighted the distinct contributions of active versus receptive music practices. Active engagement, such as singing or group music-making, was shown to foster self-expression, peer connection, and emotional release (Porter et al., 2017; Uhlig et al., 2018). In contrast, receptive approaches, such as listening to calming or instrumental music, appeared to promote relaxation and physiological down-regulation (Bong et al., 2021; Kwok, 2019). While both formats demonstrated efficacy, direct comparisons between the two remain limited, leaving open questions regarding which format may be most effective for adolescent populations.

### 4.2 Clinical vs. school-based populations

A key observation concerns the diversity of study populations. Ten studies focused on adolescents experiencing psychological difficulties, including test anxiety, trauma-related symptoms, or depressive tendencies (Alemdar et al., 2023; Zhou et al., 2025). Four studies examined adolescents with physical illnesses, such as leukemia or chronic pain (Nelson et al., 2017). Meanwhile, six studies targeted school-based, generally healthy adolescents, showing that music can also function as a preventive and supportive tool in educational settings (Ugwuanyi et al., 2020; Uhlig et al., 2018). The evidence from these school-focused studies underscores the feasibility of implementing music interventions in everyday academic environments, where anxiety related to examinations, performance pressure, and social interactions is highly prevalent.

### 4.3 Cultural adaptation and music type

A major limitation across the included studies was the lack of detail about the type of music used. Many interventions were broadly described as “relaxing music” or “classical music” without specifying the genre, tempo, or cultural relevance (Saghaee-Shahriari and Mostafazadeh, 2019). Considering that adolescents’ engagement with music is strongly influenced by cultural identity and personal preference, this lack of specificity limits interpretability and cross-cultural relevance. Future research should explore whether culturally familiar forms—such as traditional Chinese instrumental music for Chinese adolescents—can enhance the acceptability and therapeutic effectiveness of interventions. Culturally adapted interventions are vital in school settings, where student receptivity plays a key role in the success of preventive programs.

### 4.4 Mechanisms of effect

While most studies reported reductions in anxiety, relatively few explicitly examined how music produces these effects. Based on patterns observed across the included studies, several psychological pathways have been proposed in the broader literature (Chanda and Levitin, 2013; Koelsch, 2014) that may help explain adolescents’ responses to music-based interventions. These pathways are not mutually exclusive and likely operate in combination.

First, the Emotional Regulation Pathway suggests that music—especially active forms such as improvisation, singing, or group performance—provides opportunities for self-expression and emotional processing. This interpretation is consistent with studies in which active music-making was accompanied by improvements in emotion regulation or self-expression skills (Iyendo et al., 2024; Porter et al., 2017; Uhlig et al., 2018).

Second, the Physiological Down-regulation Pathway posits that music can modulate autonomic activity and induce relaxation. Evidence from several studies supports this possibility, including reductions in salivary cortisol following music intervention (Chen et al., 2019) and improvements in neurophysiological indicators of stress regulation (Park et al., 2023).

Third, the Attentional Modulation Pathway suggests that music may redirect attention away from threat-related or ruminative thoughts. This interpretation aligns with findings from interventions targeting smartphone addiction, where music functioned as an alternative attentional focus (Bong et al., 2021), and with studies in medical settings in which music reduced procedural anxiety by diverting attention (Alemdar et al., 2023; Nelson et al., 2017).

These pathways may operate simultaneously within a single intervention. For example, group drumming may involve emotional expression (emotion regulation), rhythmic entrainment (physiological regulation), and sustained focus (attentional engagement). Future research employing multimodal assessment—such as integrating physiological, behavioral, and self-report data—will be necessary to clarify the relative contribution of each pathway.

### 4.5 Integration with other therapeutic modalities

Although music alone shows promise, few studies have examined its use alongside other evidence-based practices. Combining music with mindfulness, yoga, or school-based well-being programs could potentially strengthen emotional and attentional outcomes (Zoogman et al., 2015). For instance, pairing mindfulness exercises with culturally familiar music may enhance engagement and create a more supportive environment for stress regulation in adolescents. Such integrative approaches warrant systematic investigation.

## 5 Limitations and future research

This review has several limitations. First, although a systematic search and screening process was implemented, the included studies differed markedly in their conceptual foundations and methodological approaches. Variations in intervention characteristics (e.g., music type, delivery format, session length), sample profiles, and outcome measures were substantial enough to prevent a meaningful meta-analysis. Synthesizing such heterogeneous datasets would risk generating distorted effect estimates; therefore, in accordance with PRISMA 2020 guidance, a narrative synthesis was adopted. A future meta-analysis may be feasible once a more comparable and methodologically coherent body of evidence becomes available. In addition, prospective meta-analysis—where study protocols, intervention components, and outcome measures are harmonized in advance across research teams—may provide a promising strategy to improve comparability and reduce methodological heterogeneity in this field.

Second, the overall reporting quality of the included studies was uneven. Many studies provided limited information on intervention fidelity, adherence rates, or the specific musical properties used, which restricts replicability and reduces the interpretability of intervention effects. Methodological quality indicators related to blinding should also be interpreted with caution. Due to the nature of music-based interventions, blinding of participants and therapists is generally not feasible, as such interventions require active engagement or perceptual awareness of music. Therefore, low scores on blinding items reflect an inherent limitation of the intervention design rather than poor methodological quality. In addition, few studies assessed participants’ music preferences, even though preference is known to influence engagement and emotional responses to music (Chanda and Levitin, 2013).

Third, although this review focused on adolescents, several studies included mixed-age samples where only a subset fell within the adolescent range. This may introduce ambiguity regarding age-specific effects and limit the ability to draw firm developmental conclusions.

Beyond these study-level issues, this review was also constrained by language restrictions, reliance on narrative synthesis, and the diverse range of designs and measurement tools represented in the included articles. Future research should prioritize culturally grounded music interventions, such as examining the effects of traditional or locally meaningful music styles within adolescents’ cultural contexts. This may be especially relevant in settings such as China, where traditional instrumental music carries distinctive aesthetic and philosophical meanings that may enhance acceptability and therapeutic potential. Moreover, integrating music with other complementary approaches—such as mindfulness, yoga, or school-based well-being programs—may yield synergistic benefits.

Notably, most included studies assessed outcomes immediately following intervention completion or within relatively short assessment periods, limiting conclusions regarding the long-term sustainability of intervention effects. Only a limited number of studies incorporated follow-up assessments beyond the intervention period. For example, Porter et al. (2017) evaluated outcomes at both 13 and 26 weeks post-randomization. Although broader research suggests that enriched musical experiences may have lasting cognitive and neural benefits (Hyde et al., 2009; Metzler et al., 2013), evidence specifically linking music-based interventions to sustained reductions in anxiety among adolescents remains limited. Further longitudinal research with extended follow-up periods is therefore needed.

Finally, future studies would benefit from clearer intervention protocols, broader inclusion of adolescent subgroups (e.g., healthy, subclinical, clinical, at-risk), and longitudinal follow-up incorporating both state and trait measures of anxiety to examine the persistence and stability of intervention effects over time.

## 6 Conclusion

This systematic review suggests that music-based interventions can reduce anxiety in adolescents across clinical, educational, and community settings. Both active and receptive forms of engagement demonstrated beneficial effects, although active participation generally produced broader emotional gains. These findings suggest that music may influence anxiety through multiple pathways, including emotional regulation, attentional modulation, physiological calming, and social connection. However, substantial heterogeneity exists across studies, and limitations related to reporting, sample size, and follow-up duration underscore the need for more rigorous and theoretically grounded research. Future studies should prioritize culturally relevant musical materials, investigate underlying psychological mechanisms, and explore integration with established mental health interventions. In addition, future research should incorporate long-term follow-up and both state- and trait-level measures of anxiety to better assess the durability and stability of intervention effects over time. Such efforts may expand the role of music as a culturally adaptable and accessible strategy for promoting emotional well-being among young people.

## Data Availability

The original contributions presented in the study are included in the article/supplementary material, further inquiries can be directed to the corresponding author/s.

## Footnotes

1
www.pedro.org.au

2
https://pedro.org.au/wp-content/uploads/PEDro_scale.pdf
